# A practical and systematic approach to organisational capacity strengthening for research in the health sector in Africa

**DOI:** 10.1186/1478-4505-12-11

**Published:** 2014-03-03

**Authors:** Imelda Bates, Alan Boyd, Helen Smith, Donald C Cole

**Affiliations:** 1 Department of International Public Health, Liverpool School of Tropical Medicine, Pembroke Place, Liverpool L3 5QA, UK; 2 Manchester Business School, Oxford Road, Manchester M13 9PL, UK; 3 Dalla Lana School of Public Health, Health Sciences Building, 155 College Street, Toronto M5T 3 M7, Canada

**Keywords:** Africa, Capacity building, Capacity development, Health systems, Organisational capacity, Organisational capacity development, Research capacity strengthening

## Abstract

**Background:**

Despite increasing investment in health research capacity strengthening efforts in low and middle income countries, published evidence to guide the systematic design and monitoring of such interventions is very limited. Systematic processes are important to underpin capacity strengthening interventions because they provide stepwise guidance and allow for continual improvement. Our objective here was to use evidence to inform the design of a replicable but flexible process to guide health research capacity strengthening that could be customized for different contexts, and to provide a framework for planning, collecting information, making decisions, and improving performance.

**Methods:**

We used peer-reviewed and grey literature to develop a five-step pathway for designing and evaluating health research capacity strengthening programmes, tested in a variety of contexts in Africa. The five steps are: i) defining the goal of the capacity strengthening effort, ii) describing the optimal capacity needed to achieve the goal, iii) determining the existing capacity gaps compared to the optimum, iv) devising an action plan to fill the gaps and associated indicators of change, and v) adapting the plan and indicators as the programme matures. Our paper describes three contrasting case studies of organisational research capacity strengthening to illustrate how our five-step approach works in practice.

**Results:**

Our five-step pathway starts with a clear goal and objectives, making explicit the capacity required to achieve the goal. Strategies for promoting sustainability are agreed with partners and incorporated from the outset. Our pathway for designing capacity strengthening programmes focuses not only on technical, managerial, and financial processes within organisations, but also on the individuals within organisations and the wider system within which organisations are coordinated, financed, and managed.

**Conclusions:**

Our five-step approach is flexible enough to generate and utilise ongoing learning. We have tested and critiqued our approach in a variety of organisational settings in the health sector in sub-Saharan Africa, but it needs to be applied and evaluated in other sectors and continents to determine the extent of transferability.

## Background: The problem to be addressed

Capacity strengthening in the health sector is sometimes narrowly defined as increasing the skills and technical capacity of individuals working within a variety of policy- or service-oriented programmes or organizations [1]. However, individuals do not work in a vacuum. Their skills and capacity are strongly influenced by, and influence, the institution and systems within which they operate. Specific components of the health system, for example laboratory services [2] or research infrastructure [3,4], are also important areas for capacity strengthening [1]. We therefore take an inclusive view of capacity strengthening as a process of improving individual skills, processes, and structures at the organisational level and the networks and context in which the organisation functions.

There has been substantial investment by the international health and development community in capacity strengthening in low and middle income countries. However, robust evidence to guide the design of organizational capacity strengthening programmes and measure effectiveness and value for money remains fragmented [5]. Within the published literature there is some information about methods and frameworks to guide capacity strengthening programmes but, generally, guidance about how these can be effectively operationalized is inadequate [6,7]. There are also published descriptions and case studies of operational capacity strengthening programmes [8], but these do not usually describe underpinning theories and research. There are very few published articles that bring these theoretical and practical aspects together and which describe research capacity strengthening programmes that have been explicitly designed around a strong evidence base. Challenges concerning how to use evidence to make research capacity strengthening programmes more effective have been debated at several recent international conferences [9-11]. These opportunities for face-to-face dialogue between researchers, policy-makers, and funders have resulted in general agreement on the need to better design and evaluate research capacity strengthening programmes in the health sector.

Formulating a general approach for planning research capacity strengthening in the heath sector is complex [12]. This is because the context and scale of interventions, and therefore the goals and activities, vary so much. Attempts to draw out common methods for evaluating research capacity strengthening from published papers are limited by a lack of detail in the papers about the objectives, methods, and indicators for monitoring progress [13]. The starting point for many research capacity strengthening programmes is often a ‘needs assessment’, also referred to as a gap assessment, which can be defined as ‘the systematic study of a problem or innovation, incorporating data and opinions from varied sources, in order to make effective decisions or recommendations about what should happen next.’ Gap analysis, needs analysis, and performance analysis have been used synonymously with ‘needs assessment’ although they are more accurately described as needs assessment tools.

In this paper, we describe our approach for identifying and using evidence to guide the design and implementation of health research capacity strengthening programmes. Since there are very few peer-reviewed publications evaluating the effectiveness of different approaches to capacity strengthening [13], the evidence we used included both peer reviewed publications and grey literature. The target of the capacity strengthening approach is organizations taking into account the wider health system context and the individuals who are key players in the capacity strengthening initiative. Our approach is partly based on steps implicit in the definition of a needs assessment (i.e., identification of the ‘problem’, soliciting inputs from multiple perspectives and formulating recommendations and subsequent actions). A needs (or gap) assessment starts with an explicit description of the desired outcomes which is then compared with current performance to identify the ‘need’ or ‘gap’ in performance. The first steps in our approach additionally incorporate a participatory process to agree and define the goal of the capacity strengthening intervention, and a comprehensive review of existing literature to inform a robust description of desired capacity to achieve optimal performance. These additional aspects help us to be confident that stakeholders engage with the purpose of the intervention and that existing evidence is incorporated to make the intervention more effective.

Our capacity strengthening approach comprises five practical steps for the design and evaluation of research capacity strengthening programmes, which are based on published evidence, and incorporate our own experience of applying research principles to designing and implementing a range of research capacity strengthening programmes in the health sector in Africa [14-18]. We draw on three contrasting case studies to provide a practical illustration about how to implement the steps along the pathway in order to bring about change in capacity (see Case Studies below); details of two of these studies have been published elsewhere [16,18]. The case studies represent efforts to strengthen research capacity at the organisational level in a teaching hospital, a research laboratory, and universities in Africa. These are common types of settings for capacity strengthening efforts. Drawing on our experience of designing and evaluating these interventions and programmes, we comment on the applicability of our approach to other research capacity strengthening initiatives and programmes in the health sector.

### Case studies

#### Case study 1: Laboratory systems for elimination of lymphatic filariasis in Africa [unpublished data]

##### Goal and operational focus

A university-affiliated research institution in Ghana aimed to become a West African regional centre of excellence for lymphatic filariasis. To achieve this, it needed to strengthen its role as a referral and quality assurance laboratory, and its capacity to provide on-site and outreach training across the region. This institution was part of a project network which included others in Malawi and Kenya.

##### Optimal capacity needed to achieve goal

Evidence to define optimal capacity was derived from Global Laboratory Initiative Stepwise Process towards Tuberculosis Laboratory Accreditation and adapted for NTD laboratories [19], the EFQM excellence model [20], the SIDA evaluation model of HEPNet [21], and the United Nations Development Program *Measuring Capacity* document [22]. Optimal capacity included the ability of the institution to provide training, quality assurance, and support for national lymphatic filariasis elimination programmes. This would require refurbishing a teaching laboratory, establishing new molecular techniques, and obtaining internationally recognized accreditation status.

##### Gaps in existing capacity compared to required capacity

Data collection methods included interviews, laboratory checklists, and observations of facilities. Gaps identified included inadequate teaching and laboratory facilities; lack of personnel/skills, strategic and business plans, external quality assessment and communication plans.

##### Actions to fill capacity gaps

Established quality systems and recruited quality officer, collected evidence and publicized impact of international activities; enhanced teaching and technical skills, and laboratory facilities.

##### Learning through doing and adapting the plan

Later adaptations to the plan included the need to redesign the training curriculum and to update the laboratory business plan.

##### Inputs by capacity strengthening monitoring team

Initial visits to each laboratory, pre-visit questionnaires, monthly and ad hoc Skype meetings for one year (2012–2013) to record progress and adapt plans.

##### Mechanisms for sustainability

Set up in-house laboratory training, used capacity strengthening plan to lever additional resources, established a marketable quality checking service and training programme for lymphatic filariasis control.

#### Case study 2: PhD programmes in five African universities [18]

##### Goal and operational focus

To strengthen the universities’ capacity for doctoral training programmes in five universities in Ghana, Malawi, Senegal, Tanzania, and Uganda.

##### Optimal capacity needed to achieve goal

Evidence to define optimal capacity was derived from literature concerning personal development planning for researchers, quality assurance of education, and universities’ codes of practice. Needs assessment covered policies, research facilities, admissions, supervision, research skills training, assessment, student welfare, appeals, and feedback.

##### Gaps in existing capacity compared to required capacity

Data collection methods included interviews, focus group discussions, and observations of facilities. Gaps identified included lack of PhD handbooks and of induction and research skills courses, internet access, academic meetings, local supervisors, and PhD office space.

##### Actions to fill capacity gaps

Enhanced internet access, identified dedicated PhD offices, produced PhD handbook, and trained more supervisors.

##### Learning through doing and adapting the plan

Later adaptations to plan included combining existing courses with new modules to create induction and research skills courses, and enhancing data backup systems.

##### Inputs by capacity strengthening monitoring team

Initial visits to research facilities in each university (2009), identification of capacity gaps with recommendations about actions to fill gaps, review of progress in year 3 (2012).

##### Mechanisms for sustainability

Training of local supervisors; strengthened research administration and financial infrastructure.

#### Case study 3: Improve generation and utilisation of research in a teaching hospital in Ghana [16]

##### Goal and operational focus

To generate and utilise research to ultimately improve hospital care for patients.

##### Optimal capacity needed to achieve goal

Evidence to define optimal capacity was derived from literature on effective adult learning, research processes, and institutional change management. Needs assessment covered individuals’ research skills, and institutional systems for demanding, supporting, and using research.

##### Gaps in existing capacity compared to required capacity

Data collection methods included interviews, focus group discussions, and students’ performance in assignments. Gaps identified included insufficient research skills teachers, lack of biostatistics and social science expertise, and inadequate research funds and internet access.

##### Actions to fill capacity gaps

Established research skills course and trained course facilitators; improved internet facilities; employed biostatistician; senior managers involved in demanding and utilizing research.

##### Learning through doing and adapting the plan

Early indicators included course attrition rates and examination marks; later adaptations included promotion of use of research to influence clinical practice and institutional systems, and extension of the course to two new sites.

##### Inputs by capacity strengthening monitoring team

Initial support for design of programme and monitoring indicators (2002), twice yearly (2002–2005) then once yearly (2006–2010) visits, quarterly Skype/telephone meetings (2002–2010). Now local faculty are responsible for monitoring.

##### Mechanisms for sustainability

Course financed through fees and hospital contributions; hospital unit established with responsibility for all research activities; UK award for course resulted in increased applications.

## Methods: Five-step pathway for capacity strengthening programmes

Published evidence indicates that since a key aim of any capacity strengthening programme is to promote change, such programmes should be based on an explicit theory of change [12]. For programmes concerned with strengthening research capacity, the first stage in the change pathway involves ensuring there is demand for the programme and identifying the potential stakeholders. This is followed by defining the programme’s purpose and making explicit the links between activities, outputs, and outcomes [23,24]. The theory of change also requires clarity about how the context and any underlying assumptions may influence whether the goal is achieved. Using the principles of the theory of change combined with published evidence about successful strategies for capacity strengthening [25-27], we developed a five-step approach for designing and conducting research capacity strengthening programmes. We tested our five-step approach in capacity strengthening programmes implemented across multiple countries in sub-Saharan Africa. In this paper, we use three of these programmes as case studies to illustrate how our approach worked in practice.

### Define the goal of the capacity strengthening programme

The first stage in our research capacity strengthening process was to ensure that the intervention addressed local priorities and had the potential to be viable, affordable, and sustainable. Once the intervention was broadly agreed upon, we defined the goal of the capacity strengthening intervention, as well as an overarching framework that linked the goal to activities, outputs, and outcomes. This was done in consultation with the direct beneficiaries since these were usually the major drivers of the capacity strengthening programme. These beneficiaries articulated and agreed upon a clear goal for the programme [26] and determined how much the operational focus for capacity strengthening should be extended beyond the institutions, to incorporate individuals, and the broader health system. Some programmes, operated at more than one level. During this process we encouraged beneficiaries to carefully consider the social, political, and economic context in which they worked, and other assumptions that might influence the success of their capacity strengthening plans. A wider group of stakeholders – people who had an interest in the programme but were not directly involved – were then engaged to help refine the goal and to frame more specific objectives [28]. At this point, we verified with the programme funders that the refined goal also met their requirements [27]. This was important as a lack of agreement about the goal will make it harder for capacity strengthening to be achieved, as each cadre of stakeholders might retain different expectations, thereby potentially creating an ineffective programme and acrimonious relationships.

Case study 1 provides an example of how this step worked in practice. The primary beneficiaries were laboratory managers and heads of departments and they proposed that their laboratory should become a regional centre of excellence for lymphatic filariasis. A key driver for this goal was the World Health Organisation targets for global control of neglected tropical diseases. The operational focus for capacity strengthening was the laboratory and its host research institution. Discussions with a wider group of stakeholders such as Ministry of Health programme managers and laboratory quality assessors, facilitated agreement concerning the goal, and the objectives and activities needed to achieve the goal. These included activities to enhance individuals’ skills (e.g., a course for trainers of molecular techniques) as well as processes at the organisational level (e.g., obtaining international accreditation).

### Describe the required capacity needed to achieve the goal

Our second step involved collating evidence about the optimal capacity needed to achieve the goal, such as published peer-reviewed research and grey literature including reports, guidelines, and recommendations. This detailed collation and synthesis of relevant evidence to underpin and provide rigour to the subsequent ‘needs assessment’ step is the most innovative part of our process. The evidence we sought was specific to the particular programme and organisational context. Although organisations were generally the focus of our capacity strengthening activities, we included evidence (published and grey literature, and expert opinion) that encompassed individuals, organisations, and systems because of the critical interdependencies across the three levels. For each programme, collation and synthesis of the evidence produced a long and cumbersome list which needed refinement by grouping related capacities to create a smaller set of optimal capacities. It was important not to discard any of the capacities on the list during this refinement process because this ‘optimal’ list constituted a holistic set of capacities which would act as a benchmark against which to compare existing capacity and to identify any gaps (i.e., it was the compass for the needs assessment). Capacity strengthening needs assessments that are not based on an evidence-informed and holistic benchmark may overlook important capacity gaps. Since many components of a capacity strengthening plan are interdependent, failing to address a critical gap may result in non-achievement and non-sustainability of the whole programme.

Experience has taught us that a broad and systematic trawl for relevant evidence and current good practices is essential for developing an optimal set of capacities tailored to each capacity strengthening programme. For example, in case study 2, an extensive literature search failed to identify any single instrument that could be used to evaluate all the policies and processes required by universities to run successful doctoral programmes. Therefore, we pooled relevant information from sources such as the *Code of Practice* from the UK Quality Assurance Agency [29], institutional standards for the contents of doctoral programmes, quality assurance guidelines for educational courses, recommendations concerning research skills to be acquired by doctoral students [14,30-32], a framework for managing institutional quality systems [33], and Personal Development Planning approaches for African doctoral students [unpublished data]. In our case study 2, checking the final, refined list of required capacities against the original list extracted from multiple sources ensured that we had not discarded any important ones during the process.

### Determine the existing capacity and identify any gaps compared to the required capacity

In the third step, we used the list of optimal capacities to guide data collection on capacity gaps and needs. Published evidence indicates that, because of the diversity, uniqueness, and complexity of each setting, a mix of different tools should be used when conducting needs assessments for capacity strengthening programmes [26]. We therefore developed specific data collection tools tailored to the context and project goal. As in case study 2, these tools often included an interview guide to engage stakeholders in discussion about existing capacity, capacity gaps, and challenges to strengthening capacity; a checklist based on optimal capacity, for use with programme beneficiaries to assess existing capacity against specific criteria; a list of documents to be reviewed; and an observation guide for visits to facilities. To help us analyse the data collected using these different tools, we developed a matrix to collate all the data into one table. Together, the tools were used to document existing capacity and to highlight any gaps in capacity. As no single individual, method, or document was likely to be able to provide complete and accurate information about capacity gaps, we triangulated the data collected across at least two sources and resolved any discrepancies in consultation with stakeholders. The latter process engaged a wide range of stakeholders [34,35] and was invaluable for understanding some of the reasons behind capacity gaps and for devising recommendations that we could act upon prospectively. This prospective approach contrasts with the retrospective way that resource, governance, and management gaps are usually identified [4]. Joint problem-solving with stakeholders was also important for identifying strengths, prioritizing critical capacity gaps, and transforming what evidence indicated was optimal into what was feasible and practical in the context of each programme. The gaps, reasons for the gaps, discrepancies, and resolutions, and potentially sustainable solutions for filling capacity gaps were all mapped onto a matrix and used to underpin an action plan. Examples of critical priorities, that we identified through this process in the case studies, were the appointment of a quality systems manager in case study 1 and training and mentoring for PhD supervisors in case study 2.

### Devise and implement an action plan to fill capacity gaps

The information gathered through a needs assessment has to be transformed into knowledge which is useful for decision making through reflection and sharing with others [26]. Therefore, in the fourth step, we worked with the beneficiaries of each programme to turn the list of priority capacity gaps into an action plan. The action plan was unique to each programme and had a goal, objectives, activities, and qualitative and quantitative indicators of progress. The plan named individuals responsible for actions. Based on the programme-specific theory of change, we were able to anticipate what indicators might be suitable for monitoring progress. However, it was important that each plan was regarded as flexible and able to be revised, as necessary, because we could not be sure at the outset which activities of the action plan would meet the objectives.

In many cases, the action plans contained activities with no, or minimal, cost implications, such as setting up new committees or re-allocating tasks among existing staff. Inevitably, some activities to strengthen research capacity required resources to cover, for example, the cost of training, or specific equipment. In these cases, the beneficiaries drafted budgets to negotiate with their funders and institutions to cover these costs. Due to the explicit buy-in of stakeholders and the evidence-informed approach, the plans and budgets had sufficient legitimacy to be used as advocacy tools for mobilising support. For example, the systematic identification of poor internet access as a priority capacity gap for African universities (case study 2) enabled university authorities to persuade a group of international funders to jointly contribute to upgrading internet access for postgraduate students.

### Learn through doing; adapt the plan and indicators regularly

**Figure 1:**
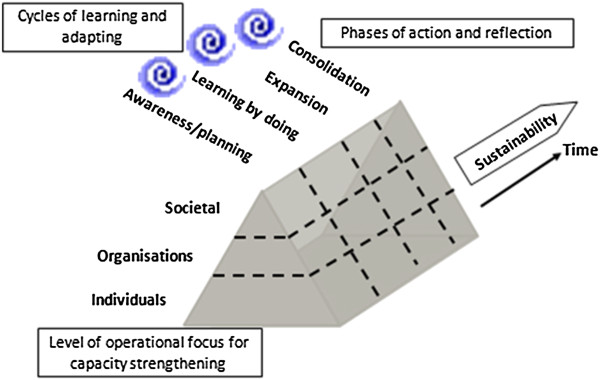
**Concept for designing and evaluating capacity strengthening programmes **[5]**.**

The process of strengthening capacity must be iterative and flexible [12]. This allows the plan, activities, and indicators to be revised as processes, relationships, structures, and agendas change. In capacity strengthening programmes, organisations typically move along a change pathway through learning cycles of action, reflecting on the changes, as they happen, and the indicators documenting them, and identifying new priorities. This process ensures that progress can be monitored, that decision-making is auditable, and that capacity is strengthened (See the five-step pathway below). We used learning cycles of action and reflection to inform revisions to the plans and indicators (Figure 1). As our capacity strengthening programmes developed, they passed through four overlapping phases – awareness, learning-by-doing, expansion, and consolidation [15]. With each phase, the programmes became more mature, some capacity gaps were filled, and new priorities emerged. We regularly re-visited the monitoring indicators because, although helpful up to that phase, indicators had to become more sophisticated as the programme matured, reflecting the increasing complexity of the programme [15]. For example, in case study 3, early indicators included course attrition rates and examination marks, whereas later indicators included evidence that research had influenced clinical practice and institutional systems [16,17].

#### Five-step pathway for designing health research capacity strengthening programmes

1. Define the goal of the capacity strengthening project

This necessitates harmonising the expectations and objectives of the most critical stakeholders including developing country partners, people involved in ensuring sustainability of the activities in the long term and the funding organisation.

2. Describe the required capacity needed to achieve the goal. This will require a search for the best evidence to describe the ‘optimal’ capacity collated from, for example, peer-reviewed published papers or expert groups, including evidence from outside the health sector.

3. Determine the existing capacity and identify any gaps compared to the required capacity. The evidence from step two is formatted into a set of qualitative and quantitative data collection tools to identify existing capacity and capacity gaps. Data is collected from stakeholders with different perspectives; discrepancies are highlighted and resolved through further discussion.

4. Devise and implement an action plan to fill the gaps

The prioritised list of capacity gaps is transformed into an action plan which includes objectives, activities, deliverables and monitoring indicators, and measures to facilitate sustainability.

5. Learn through doing; adapt the plan and indicators regularly. Results from experimentation and learning, and regular discussions with those responsible for monitoring progress are used to refine the plan. Progress indicators become more sophisticated as the programme matures and capacity is strengthened.

#### Planning for sustainability

The definition of sustainable capacity varies from programme to programme and needs to be agreed upon by the primary beneficiaries at the outset. Sustainability may mean, for example, that the programme activities are incorporated into the structures of the original organization, that they are integrated into another institution, or that the programme itself becomes an autonomous agency such as an independent charitable organisation. In a recent analysis of sustainable programmes in Africa, we demonstrated that, typically, sustainability meant that a programme had achieved financial independence and local autonomy in decision-making (see Enablers and challenges for sustainably strengthening health research capacity below). Therefore, in our capacity strengthening programmes, strategies for promoting the sustainability of ongoing activities, and their accompanying indicators, were woven into the objectives, action plan, and monitoring approach from the outset. For example, in case study 1, external accreditation of the laboratory was deemed essential for it to become a regional reference centre underpinned by a viable business strategy, so all the activities needed to achieve accreditation were reflected in the laboratory’s capacity strengthening plan from the outset. Incentives to retain staff, through further education or salary adjustments has been another key strategy which health research organizations have employed to promote sustainability [36]. In case study 3, the contentious decision to charge course fees, to students pursuing a professional course in research skills, eventually contributed to the financial security of the course and concomitantly motivated students to complete the course.

#### Enablers and challenges for sustainably strengthening health research capacity [15]

##### Enablers associated with sustainability

● Early engagement of stakeholders and explicit plans for sustaining efforts

● Ongoing learning and quality improvement cycles

● Investment in core resources (people, funds, committees, systems)

● Institutionalisation of new capacity

● Evidence of problem solving, decision-making and innovation

##### Challenges to achieving sustainability

● Turnover of staff and stakeholders

● Embedding new activities into existing systems

● Securing funding

● Influencing policy and programs

## Results

### Case studies: comparison and critique of our five-step approach for design, implementation, and monitoring of health research capacity strengthening programmes

#### Defining the goal

Although the three case studies had different goals – strengthening research skills (case study 3), strengthening laboratory systems and skills (case study 1), and providing PhD training/skills development (case study 2) – they all focused primarily on capacity strengthening at the institutional level. Individual skills training was complementary, in keeping with the close interdependence between the different levels at which capacity strengthening can occur (i.e., individual, institutional, and national/international levels). Paying attention to the skills of individuals is important even if the main focus of a programme is to strengthen institutional systems and processes.

#### Identifying optimal capacity

The availability of resources needed to inform our identification of optimal capacity for each of the programmes was highly variable. For example, there were plenty of resources describing the requirements for a laboratory to achieve accreditation (case study 1). These were helpful in determining the optimal capacity needed by laboratories for them to function as centres of excellence. Some of these requirements were already regarded as ‘gold standards’ by many of the laboratory managers involved in the programme. Information to inform optimal capacity needed to manage PhD programmes (case study 2) was less easily accessible and was drawn from a diverse sources ranging from guides for PhD skills training programmes to university codes of practice. A lack of Africa-based documentation meant that almost all of this information had been produced by wealthy countries and was therefore not necessarily entirely relevant or applicable to the countries involved in the programme. Resources used to inform optimal capacity needed by a teaching hospital to promote research (case study 3) included adaptations of research skills training programmes, and frameworks used for enhancing quality management systems in organisations.

For each of the case studies, there were iterative consultations between all those involved so that the information used to guide the programmes and describe optimal capacity was adapted to make it appropriate for the local context and programme goal.

#### Identifying capacity gaps

Similar methods were used, across the case studies, in working with beneficiaries to identify their critical gaps as compared to the optimal capacity, described in step 2. Through this process we learnt several important lessons. The interviews with stakeholders needed to be informal, ‘conversational’ style and non-judgemental, to help stakeholders talk openly about strengths and weaknesses. Involving a diverse range of stakeholders is a very powerful way of incorporating multiple views and gaining a deep understanding of the situation. However, this data needs to be managed carefully as it may reveal discrepancies which need to be followed up. Resolving these discrepancies may require re-visiting areas of controversy among multiple stakeholders. We learned to take a very flexible approach to sampling, using a snowballing technique or referral samples, in order to capture all relevant stakeholders. We were also particularly careful to consider when the use of focus group discussions was appropriate and how to be sensitive about the composition of these groups. For example, we decided not to include both laboratory managers and institution directors in focus group discussions in case study 1 because the power dynamics between them meant that junior laboratory staff would be less likely to tell us their own views about strengths and weaknesses, in an open and honest way, if their superiors were present. Knowing that staff may be hesitant about criticising their workplace, we invested time, during initial meetings, in building up trust and involving staff in a partnership with us to collect data on capacity gaps.

#### Actions to fill capacity gaps

Although the types of actions varied across the case studies because of the different goals, the action plans were all developed, refined, and modified in partnership with beneficiaries and others in their institutions. The plans were owned by the institution and the actions were derived directly from the capacity gaps that had been identified by stakeholders. An additional, and unforeseen benefit of this high level of institutional ownership, was that, in all three case studies, the primary beneficiaries found the action plans helpful in leveraging further support with their institutions or for applying for additional, external funding.

#### Consolidation and sustainability of new capacity

All three programmes described in the case studies now have established systems, processes, and capacity as a result of the interventions. The level of input from our capacity strengthening team after the action plans had been introduced varied from less than yearly contact (case study 2) to every 1–3 months (case studies 1 and 3). It was partly influenced by the support available to implement the plans. In general, over the course of 1–3 years, the inputs from our team gradually reduced as the institutions and stakeholders developed confidence and expertise in revising their action plans. Within these case studies, there are several examples of how stakeholders are now able to autonomously identify and address new capacity strengthening challenges. For example, laboratory managers (case study 1) have applied our capacity strengthening approach to develop their own regional network; university staff (case study 2) are now using this approach to strengthen institutional research administration and financial systems; the hospital-based research skills course (case study 3) is locally-run and self-sustaining, and research findings from the students’ projects are being used to improve health services.

## Discussion

We have used a variety of evidence to develop a systematic five-step approach to designing capacity strengthening programmes. Using three case studies as examples of how we have implemented this approach in practice, we have shown how it can be applied in different contexts to assist programmes in achieving a variety of goals. By comparing the challenges we encountered in applying this approach and by identifying commonalities among the case studies, we have been able to draw out some generic lessons. In particular, we have demonstrated the importance of considering the interdependence of the different levels of capacity strengthening activities (i.e., individual, institutional, national/international), of conducting a thorough review and collation of available evidence to inform optimal capacity, of gaining trust and engaging stakeholders throughout the process, and of reducing external support over time to match increases in local capacity and promote sustainability.

Our approach for conducting effective capacity strengthening does not start with a needs assessment and capacity strengthening plan alone. Rather it begins by working with stakeholders to make explicit the programme components needed to develop the optimal capacity required in keeping with the agreed upon goal for each programme. Although some guidelines mention a preliminary start-up phase for defining the purpose and scope of the capacity strengthening programmes, the emphasis is often on a narrowly construed needs assessment [26]. In the start-up phase of the capacity strengthening programmes described herein, we worked with key beneficiaries to list all the components of systems, staff, skills, and tools [37] needed to achieve the desired capacity. Using this list gave us confidence that we were unlikely to miss any critical capacity gaps. The in-depth understanding of what optimal capacity looked like in each context and for each goal enabled us to carefully consider what data collection methods would be appropriate, and to identify and develop evidence-based data collection tools. Each data collection tool had a pre-defined purpose and was tailored to specific stakeholder groups. Capturing all the data on a matrix enabled us to rapidly scrutinise information from multiple stakeholders. The matrix also facilitated identification and prioritisation of gaps and potential responses. It helped us to devise an action plan that would lead to sustainable strengthening of capacity.

### Limitations

We recognize that health research capacity strengthening systems have indistinct boundaries and so we may have overlooked some factors that could have influenced the effectiveness of the programmes. To mitigate this risk, we discussed and refined every component on the basis of inputs from beneficiaries and other stakeholders [38]. We ensured that the objectives and activities were relevant for achieving shorter and longer term goals, that the activities were planned on a realistic timeline, and that monitoring indicators were assigned to each activity. We tried to ensure that the indicators did not require over-burdensome data collection, yet still satisfied funders’ accountability requirements [26]. Our intensive consultation and openness to options at the start of the programme cultivated trust and local ownership. These, together with our external support, permitted the type of light touch management needed to adapt plans and indicators as the programmes matured.

### Principles, theories, and models that have informed our approach

Our philosophy for effective capacity strengthening is built on three principles which have been derived from a variety of published evidence: start small, build on existing capacity, and foster trusting and respectful partnerships [28]. Our systematic approach has been informed by established theories and methods. For example, the steps along the pathway to achieving sustainable capacity strengthening are based on an explicit theory of change [39]. The stakeholder interviews use the established qualitative method ‘informal conversational interview’; with this approach, rounds of interviews are conducted, each building on themes identified in previous interviews, to expand on emerging gaps, to elaborate and resolve discrepancies, and to move in new directions [40]. Our approach to conceptualising capacity strengthening programme development (Figure 1) operates at three levels (individuals, organisations, societal) because interventions at one level will inevitably influence, and be influenced by, factors at other levels. The approach incorporates opportunities for reflecting on how change occurs, which is important for strategic and methodological clarity about how to continually develop capacity [41]. Our approach also extends the focus of capacity strengthening beyond just technical matters and promotes more effective and dynamic relationships among different stakeholders [27]. We acknowledge that capacity strengthening may well produce technical and managerial changes yet cultural shifts such as changing attitudes, promoting collective decision making and networking, and facilitating dialogue across stakeholder groups are also important, as exemplified in a study about building research capacity in Zambia [42].

## Conclusions: Feasibility of adopting our approach

Our approach for designing and evaluating health research capacity strengthening programmes has been tested and re-worked in a variety of health organisations in sub-Saharan African countries to meet the needs of different funding agencies. Although the methods we describe have been used primarily for capacity strengthening for health research and service provision, they may be applicable outside of these contexts. Our approach does, however, depend critically on a committed multi-disciplinary team, and on having enough time and resources to enable the goal, objectives, activities, and indicators to be agreed upon and planned collaboratively [42]. We recognize that in many low income countries the limited expertise in evaluating capacity strengthening efforts may need to be addressed [43] before our approach can be implemented without external support.

Nevertheless, our strategy of encouraging those involved in health research capacity strengthening to set goals early in the project cycle and to define what capacity would be optimal and feasible, has proved to be an adaptable and feasible approach in the challenging contexts encountered in low income countries in sub-Saharan Africa. We encourage others to consider applying our methods and approaches in other sectors and continents to determine their usefulness and the extent of their transferability.

## Competing interests

The authors declare that they have no competing interests.

## Authors’ contributions

IB conceived the idea for the paper and has been involved in the case studies included in the paper; she produced the first draft and contributed to all subsequent drafts of the paper. AB provided evidence for the frameworks discussed in the paper and identified the model shown in the figure; he contributed to all drafts of the paper. HS provided evidence to support several of the methodologies in the paper including the logic models and informal conversational interviews; she contributed to all drafts of the paper. DCC put the idea, models, and practices outlined in the paper into the global context and provided a critical international perspective; he contributed to all drafts of the paper. All authors contributed intellectually to the paper by sharing and debating their ideas which helped us explain and justify our capacity strengthening approach, and by providing relevant supporting evidence. All authors have seen and approved the final version of the paper.
